# Three-Dimensional Deep Learning Normal Tissue Complication Probability Model to Predict Late Xerostomia in Patients With Head and Neck Cancer

**DOI:** 10.1016/j.ijrobp.2024.07.2334

**Published:** 2024-08-13

**Authors:** Hung Chu, Suzanne P.M. de Vette, Hendrike Neh, Nanna M. Sijtsema, Roel J.H.M. Steenbakkers, Amy Moreno, Johannes A. Langendijk, Peter M.A. van Ooijen, Clifton D. Fuller, Lisanne V. van Dijk

**Affiliations:** * Department of Radiation Oncology, University Medical Center Groningen, University of Groningen, Groningen, The Netherlands; † Department of Radiation Oncology, The University of Texas MD Anderson Cancer Center, Houston, Texas

## Abstract

**Purpose:**

Conventional normal tissue complication probability (NTCP) models for patients with head and neck cancer are typically based on single-value variables, which, for radiation-induced xerostomia, are baseline xerostomia and mean salivary gland doses. This study aimed to improve the prediction of late xerostomia by using 3-dimensional information from radiation dose distributions, computed tomography imaging, organ-at-risk segmentations, and clinical variables with deep learning (DL).

**Methods and Materials:**

An international cohort of 1208 patients with head and neck cancer from 2 institutes was used to train and twice validate DL models (deep convolutional neural network, EfficientNet-v2, and ResNet) with 3-dimensional dose distribution, computed tomography scan, organ-at-risk segmentations, baseline xerostomia score, sex, and age as input. The NTCP endpoint was moderate-to-severe xerostomia 12 months postradiation therapy. The DL models’ prediction performance was compared with a reference model: a recently published xerostomia NTCP model that used baseline xerostomia score and mean salivary gland doses as input. Attention maps were created to visualize the focus regions of the DL predictions. Transfer learning was conducted to improve the DL model performance on the external validation set.

**Results:**

All DL-based NTCP models showed better performance (area under the receiver operating characteristic curve [AUC]_test_, 0.78–0.79) than the reference NTCP model (AUC_test_, 0.74) in the independent test. Attention maps showed that the DL model focused on the major salivary glands, particularly the stem cell-rich region of the parotid glands. DL models obtained lower external validation performance (AUC_external_, 0.63) than the reference model (AUC_external_, 0.66). After transfer learning on a small external subset, the DL model (AUC_tl, external_, 0.66) performed better than the reference model (AUC_tl, external_, 0.64).

**Conclusion:**

DL-based NTCP models performed better than the reference model when validated in data from the same institute. Improved performance in the external data set was achieved with transfer learning, demonstrating the need for multicenter training data to realize generalizable DL-based NTCP models.

## Introduction

Normal tissue complication probability (NTCP) models predict the risk of developing radiation-induced toxicities. These predicted risks could guide treatment decision-making, for example, model-based selection for advanced treatments such as proton therapy,^1^ or guide the radiation plan optimization process. One of the most common radiation-induced toxicities in patients with head and neck cancer (HNC) is xerostomia (dry mouth),^2,3^ which has a significant impact on a patient’s quality of life, because it can severely hamper eating, swallowing, tasting, and speech ability. Hence, accurate prediction is crucial to facilitate personalized radiation therapy approaches to prevent xerostomia.

Historically, NTCP models were Lyman-Kutcher-Burman models that are based on uniform equivalent doses of a single organ-at-risk (OAR).^4–7^ More recent NTCP models for xerostomia are multivariable and typically based on a number of discrete dose parameters (eg, mean doses) of salivary glands and baseline xerostomia scores.^8,9^ These models have been shown to outperform the Lyman-Kutcher-Burman model design.^10^ In multivariable and Lyman-Kutcher-Burman models, the planned radiation dose administered to the salivary glands is reduced to a single value, whereas, in reality, radiation-induced salivary gland damage is a 3-dimensional (3D) phenomenon.^11^ Previous studies showed differences in the spatial effect of parotid gland dose on xerostomia development by using voxel mapping.^11–13^ This indicated that xerostomia prediction models might be improved if they could use the full 3D radiation dose information. Additionally, it was demonstrated that xerostomia prediction could be improved with image features of the salivary glands in combination with dose parameters^8,14,15^ or with dosiomics,^16^ exemplifying that patient-specific image characteristics can improve the prediction of xerostomia.

Deep learning (DL) models, specifically convolutional neural networks, can extract relevant features from 3D information directly. These models can be trained to recognize the individual patient’s anatomy and link this to the spatial information of the radiation dose distribution. The feasibility of using DL for toxicity prediction has been piloted previously, but the data sets were limited. In contrast, Men et al^17^ showed that xerostomia could be predicted by using the entire 3D dose distribution, multiorgan, and image information as input. Their residual-based DL model outperformed a one-dimensional logistic regression model in predicting late xerostomia in the RTOG-0522 HNC patient cohort.^17^ However, the prediction of xerostomia with DL could be further improved by including clinical variables in the DL modeling process and by considering more salivary-related OARs, because both have been shown to be important predictors.^8,9,15^ Moreover, this current study also investigates alternative DL architectures, explores the potential of transfer learning to accommodate data from an external medical center, implements a comprehensive model evaluation that conforms to the conventional NTCP modeling standards, and explores attention maps to “open the black DL-box,” identifying focus regions used for the DL xerostomia predictions. Finally, to pursue clinical implementation of the DL-based NTCP model, development was performed with a large-scale prospective toxicity data set collected through standard follow-up in patients treated with the current standards in radiation therapy.

Consequently, this study aimed to improve the prediction of moderate-to-severe xerostomia 12 months postradiation therapy (Xer_12m_) with a 3D DL-based NTCP model compared with a conventional NTCP model^8^ developed in a large HNC patient cohort. The hypothesis is that by considering the 3D dose distribution, computed tomography (CT) image, and salivary-related OAR segmentations, in combination with clinical variables, late xerostomia risk can be better estimated.

The source code has been made publicly available at https://github.com/PRI2MA/DL_NTCP_Xerostomia

## Methods and Materials

### Patients and treatment characteristics

Patients with proven squamous cell carcinoma who received radiation therapy with curative intent at the UMC Groningen between April 2007 and February 2021 were included in model development and testing. Patients were excluded if they had a skin tumor, received previous HNC irradiation, a fraction dose >2.4 Gy, had no primary radiation therapy, had incomplete irradiation, had palliative irradiation, had a history of leukemia, had induction chemotherapy, or had no toxicity score at the start or 12 months postradiation therapy. The UMC Groningen cohort was split into a training and cross-validation set (85%) for model development and an independent test set (15%) for model evaluation. The split was stratified on contrast-enhanced CT scans, CT metal artifacts, treatment modality (photons or protons), and tumor location.

For external validation, patients from the University of Texas MD Anderson Cancer Center (2015–2020) were included in the https://github.com/PRI2MA/DL_NTCP_Xerostomia registry, who otherwise met the same inclusion criteria.

Patients typically received a primary tumor dose of 66 to 70 Gy in 6 to 7 weeks with either intensity modulated radiation therapy, volumetric-modulated arc therapy, intensity modulated proton therapy, or 3D conformal radiation therapy. In all cases, the parotid glands were spared as much as possible.^18,19^ Refer to references^15,19^ for more radiation therapy protocol details.

### NTCP endpoint

The NTCP endpoint was patient-rated moderate-to-severe xerostomia at 12 months following radiation therapy (Xer_12m_). Data from UMC Groningen patients were prospec-tively collected as part of a standardized follow-up program,^9,14,20^ where “moderate-to-severe” was defined as the highest 2 scores on the 4-point Likert scale (“none,” “little,” “moderate,” and “severe”) of the EORTC QLQ-H&N35 questionnaire. For MD Anderson patients, the xerostomia scores were prospectively collected from MD Anderson Symptom Inventory-Head and Neck Module questionnaires with an 11-point scale (0–10).^21^ For this study, “moderate-to-severe” was defined as scores 6–10.

### Reference NTCP model

The reference NTCP model was a multivariable logistic regression model recently published by van den Bosch et al,^8^ because this is currently the most comprehensively developed conventional xerostomia model available. The input variables were baseline xerostomia score (“none,” “little,” and “moderate-to-severe”) and parotid and submandibular glands’ mean dose. Refer to Supplementary Material E1 for more details. The reference model was refitted and cross-validated in the current cohort.

### Data preprocessing and augmentation

**Fig. 1.**
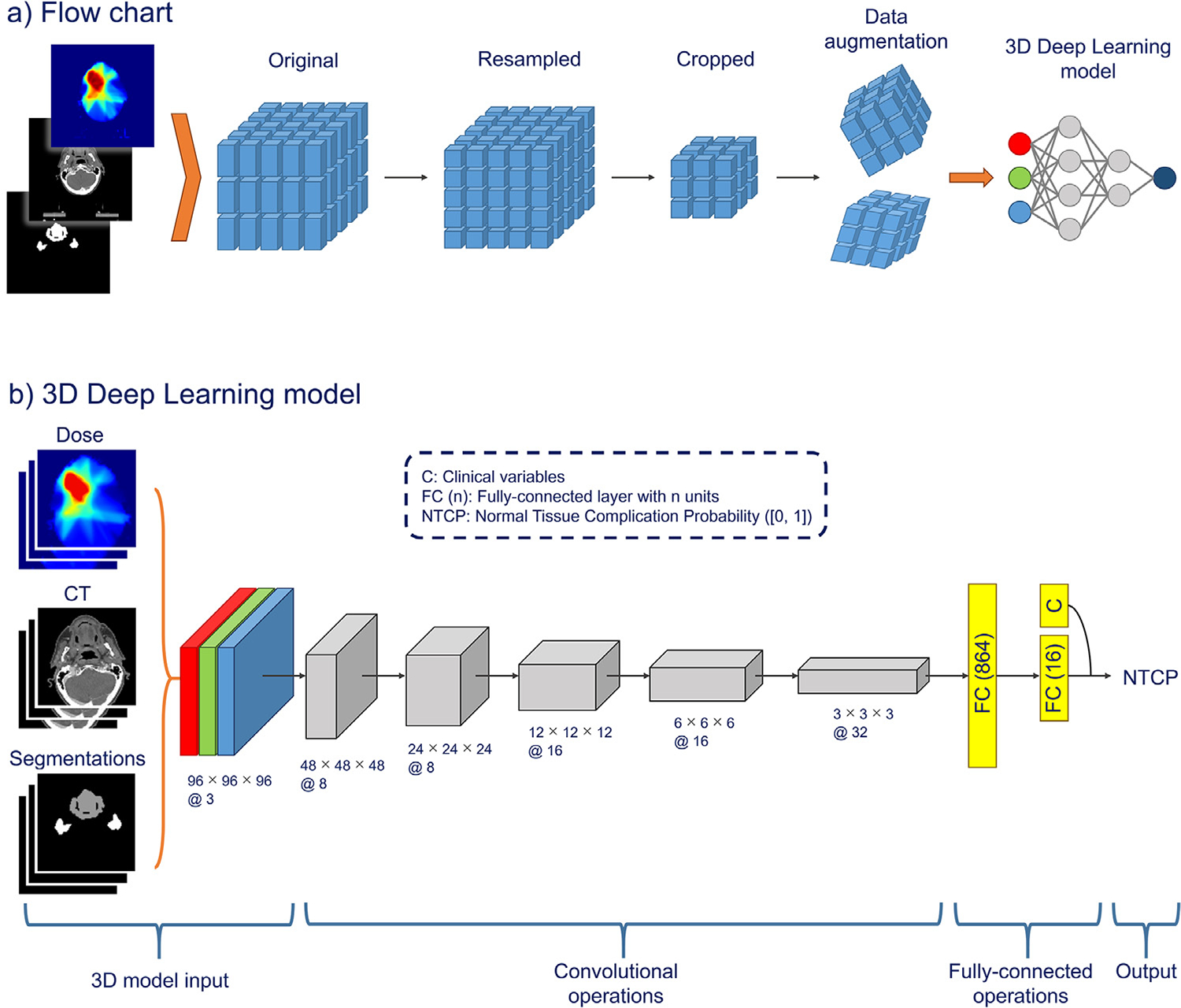
(a) Flow chart of data preprocessing and modeling: the dose distribution, CT scans, and organ-at-risk segmentations were resampled, cropped, and augmented; (b) schematic overview of the deep convolutional neural network (DCNN) and ResNet architecture. The model input was the dose, computed tomography (CT), and segmentations plus the clinical variables (c) (baseline xerostomia score, sex, and age). The output was Xer_12m_. Gray block denotes a model block with “d × h × w@c” referring to the depth × height × width with c number of channels.

An overview of the data/image preprocessing is depicted in Figure 1a, and for a detailed description, refer to Supplementary Material E2. In summary, the nominal 3D planned dose distribution, the planning CT scan, and the OAR segmentations were resampled to an isotropic voxel spacing of 2 × 2 × 2 mm^3^ (depth × height × width) and cropped to a fixed 200 × 200 × 200 mm^3^ image size, guided by the outer boundaries of the segmentations of salivary glands, cricoid, and thyroid. The diversity of the data set was enhanced by applying random 3D data augmentation techniques (ie, cropping, flipping, translation, zooming, and rotation) with a probability of 0.5 during model training.

### DL architectures

The DL model input was aligned 3D dose distribution, CT scans, and OAR segmentations (buccal mucosa, oral cavity, and salivary glands) as an image with 3 channels (3 × 96 × 96 × 96) and clinical variables (baseline xerostomia score, sex, and age). The channel-wise stacking of the aligned dose distribution, CT scans, and segmentations enables the DL models to analyze local input regions and identify patterns in dose distribution with respect to anatomic structures such as the parotid and submandibular glands. Additionally, segmentations guided the DL models and emphasized the significance of dose distribution in the OARs.

The following 3 DL architectures were implemented: a common deep convolutional neural network (DCNN)^22^, the state-of-the-art EfficientNetV2-S model,^23^ and the well-known residual neural network (ResNet).^24^ A schematic overview of the DCNN and ResNet architecture is depicted in Figure 1b. The clinical variables were concatenated to the last fully connected layer before the prediction unit (“NTCP”). The cross-entropy loss function, starting learning rate of 0.0001, and batch size of 8 were used. Refer to Supplementary Material E3 for details on the hyperparameter tuning process, DL architectures, and training procedures.

The models were trained using a stratified 10-fold cross-validation approach; an average ensemble prediction design was deployed from the resulting 10 models. The data augmentation and modeling were implemented using Project MONAI 0.9 in PyTorch 1.10.^25,26^

Finally, the improvement of DL in the external set by transfer learning was tested. For transfer learning, DL models were initialized with pretrained model weights obtained from the InstituteX_EU training set.

### Statistical evaluation

The area under the receiver operating characteristic curve (AUC) was the primary evaluation metric of the model performance. In addition, the models were evaluated in terms of the Brier score and Nagelkerke’s R^2^.^27^ Calibration curves were generated to evaluate the goodness-of-fit. The average cross-validation and ensemble performance on the independent test and external validation set were reported.

### DL input modality dependency

The importance of each input modality (dose, CT, OAR segmentations, and clinical variables) was tested by replacing the input of one of the modalities with zeroes. Additionally, the impact of CT metal artifacts on the model performance was assessed by testing the best DL model in subsets of the data classified on the level of CT metal artifact: “no,” “little/medium,” and “heavy” (Supplementary Material E4). Two subsets were considered: patients with (1) “no” and “little/medium” and (2) only “no” metal artifacts. The models were retrained in these subsets because of differences in data set composition, but no transfer learning was applied. The best-performing DL model was used for these analyses.

### Looking inside the black box

Attention maps visualize the regions the DL model focuses on when making its prediction (Supplementary Material E5).^28^ A 3D attention map using the Grad-CAM++ algorithm^29,30^ was projected onto the input data for each patient. These maps allow the identification of regions in the input data that are important for Xer_12m_ prediction.

## Results

### Patients and treatment characteristics

The InstituteX_EU cohort included 897 patients with HNC, which was split into a training and cross-validation set (n = 759) and an independent test set (n =138). The external validation cohort included 311 InstituteX_US patients. Patient characteristics were significantly different between the InstituteX_EU and InstituteX_US cohorts for all patient, tumor, and treatment characteristics (Table 1).

### DL model performance compared with reference NTCP model

The reference NTCP model performed well in the current cohort (AUC_cross-validation_, 0.75, 95% confidence interval [CI], 0.72–0.78), which is comparable with the performance reported by van den Bosch et al^8^ (AUC_validation_, 0.72). All DL models, which were based on the 3D information and clinical variables, performed significantly better (AUC_cross-validation_, 0.78–0.79; DeLong’s test *P* value, .00–.04) than the reference model (Table 2). The DCNN showed the best performance (AUC_cross-validation_, 0.79 [0.76–0.82]) of the DL architectures, but the performance of the EfficientNetV2-S (AUC_cross-validation_, 0.78 [0.75–0.81]) and ResNet (AUC_cross-validation_, 0.78 [0.75–0.81]) was comparable. During hyperparameter tuning, the optimal number of weights for the DCNN and ResNet was 140,000 to 150,000. The AdaBound optimizer^31^ and the cosine learning rate scheduler with warm restarts every 40 epochs were the most optimal.^32^ Moreover, for the best DL prediction performances, the segmentation voxel value for salivary glands was found to be 1, whereas for buccal mucosa and oral cavity, this was 0.1. The level of data augmentation had a substantial impact on the model performance; nevertheless, the optimal level varied between models. For more hyperparameter tuning results, refer to Supplementary Material E3.

**Fig. 2.**
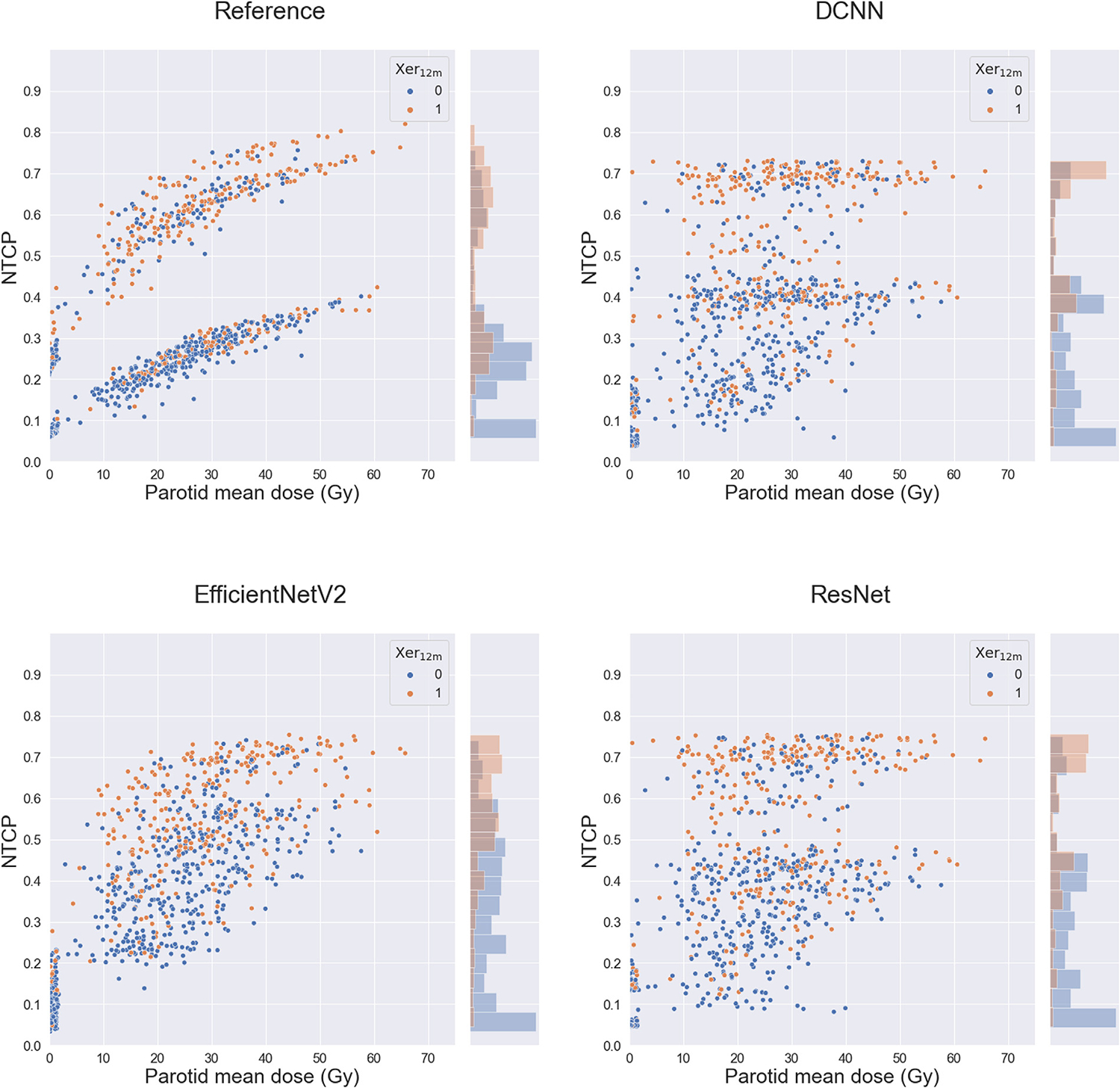
These scatterplots display the relationship between parotid mean dose (in Gy) and normal tissue complication probability (NTCP) value for all models. Patients who experienced moderate-to-severe xerostomia 12 months postradiation therapy are represented by orange, whereas the remaining patients are represented by blue. The accompanying histogram illustrates the distribution of the NTCP values.

In the independent “never-seen-by-the-model” test set, the DL models showed stable model performance (DCNN: AUC_test_, 0.79 [0.71–0.86]; Table 2). The DL models performed better than the reference model (AUC_test_, 0.74 [0.66–0.83]) in the test set, with the DCNN significantly out-performing the reference model (DeLong’s test *P* value = .04). Calibration curves of the DL models showed good goodness-of-fit in the test set (Supplementary Materials E6). Additionally, Figure 2 shows the NTCP values against the parotid glands’ mean dose for all models, illustrating a relation between predicted xerostomia probability and parotid glands’ dose. Two distinct clusters can be observed for the reference model, which was led by the baseline xerostomia score; these clusters were less profound for the DL models. Although the DL models show less reliance on the parotid glands’ mean dose, there is still a relation visible, and they have more accurate predictions than the reference model for patients with xerostomia with low parotid glands’ mean dose. Moreover, the histograms accompanying the scatterplots in Figure 2 reflect that the DL models can better discriminate the events from nonevents than the reference model.

Both the reference model (AUC_external_, 0.66 [0.59–0.73]) and the DL (DCNN: AUC_external_, 0.63 [0.55–0.70]; Table 2) performances were lower in the external validation than in the internal sets (ie, cross-validation and independent test). Moreover, external prediction performance was better in the reference model than the DL models, although not statistically significant (DeLong’s test *P* value = .37). As a subsequent analysis, transfer learning was applied to 100 randomly selected MD Anderson patients; the remaining 211 external patients were used for testing. The transfer-learned DL models showed improved performance (DCNN: AUC_external_, 0.66 [0.57–0.74]) compared with the reference model (AUC_external_, 0.64 [0.55–0.72]) in the new external set (DeLong’s test *P* value = .64). Transfer learning on different external subset sizes was evaluated for the DCNN: the performance was better when using more external patients and performed better than the reference model when using a minimum of 100 patients, although not significant (DeLong’s test *P* value, .47–.97) (figure 5 in Supplementary Material E7). The best performance was achieved when 200 patients were used for transfer learning (DCNN: AUC_external_, 0.69 [0.58–0.80]; reference: AUC_external_, 0.66 [0.54–0.77]).

### DL input modality dependency

**Fig. 3.**
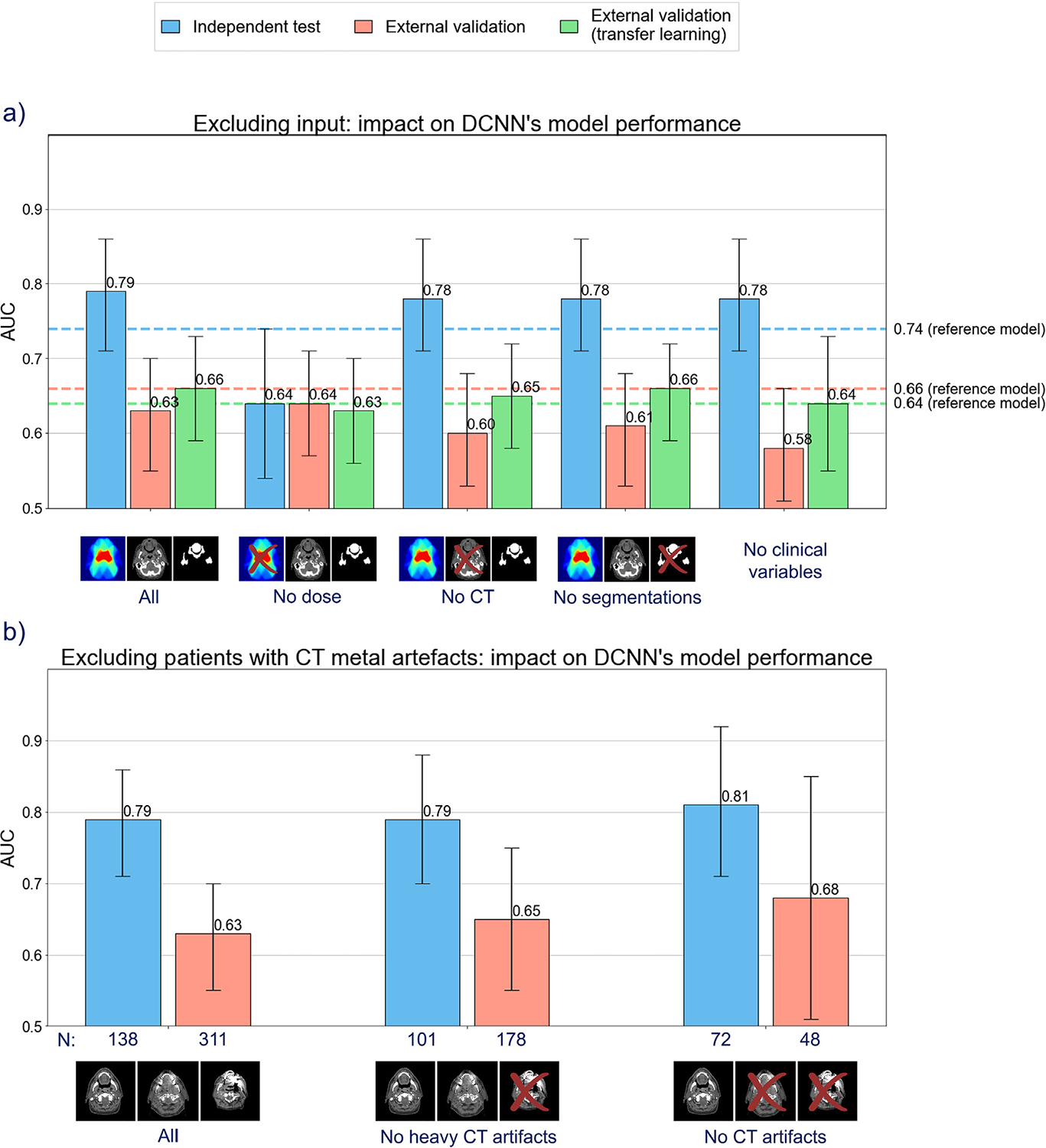
Deep convolutional neural network (DCNN) results of excluding (a) input modality and (b) patients with metal artifact computed tomography (CT) scans (where N denotes the number of patients of that data subset). The error bar indicates the 95% confidence interval.

The models’ reliance on the input data was tested by omitting each input modality independently (Fig. 3a). The predictive performance of the DCNN in the independent test set decreased substantially when dose distribution was omitted as input (AUC_test_ from 0.79 [0.71–0.86] to 0.64 [0.54–0.74]), whereas the performance decreased slightly when either the CT, the OAR segmentations, or the clinical variables were omitted (AUC_test_, 0.78 [0.71–0.86]). Nevertheless, in the external validation cohort, omitting clinical variables showed the largest reduction in predictive performance. After transfer learning, the DL model without the dose or clinical variables showed again lower performance compared with the full DL model.

Figure 3b demonstrates that training the DCNN on a cohort without metal artifact CT scans showed the best performance. The removal of “heavy” artifact scans alone did not show a difference in the independent test performance; however, an increase in performance was seen in the external data set (refer to Supplementary Material E8 for all DL results).

### Looking inside the black box

**Fig. 4.**
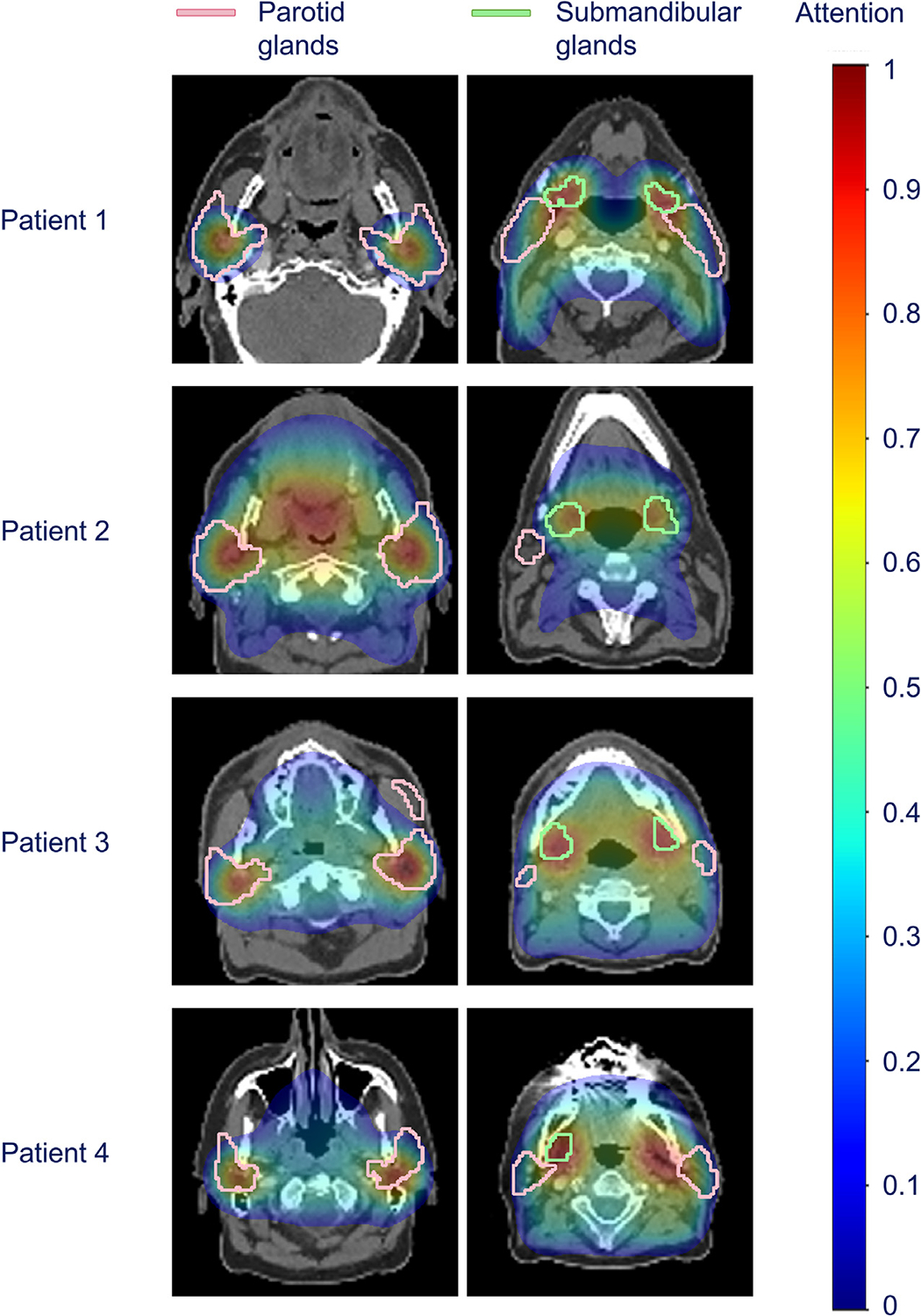
Deep convolutional neural network’s (DCNN) attention maps for 4 patients (each row) were overlaid on the computed tomography images. The red attention regions indicate that the model’s prediction was highly affected by those regions, whereas the blue attention regions indicate little impact. The pink and green contours indicate the parotid and submandibular glands, respectively.

The DCNN’s attention maps show a high focus on the parotid and submandibular glands for the vast majority of patients (Fig. 4), indicating the importance of this region in predicting Xer_12m_. Moreover, the posteriorly adjacent to the mandible in the parotid gland depicted a higher degree of attention, which corresponds to the area that contains the largest number of stem cells.^33^ Occasionally, the attention maps showed a focus on the oral cavity, and for patients with laryngeal cancer, the attention maps showed more often high attention in the high-dose region in the larynx.

## Discussion

The performance of the 3D DL-based NTCP models was enhanced compared with the reference NTCP model in predicting Xer_12m_ when tested on an independent data set of the same institution. This suggests that the DL models can leverage more sophisticated data features from the complex relationships between the entire 3D dose, CT, OAR segmentations, and clinical data, beyond the discrete dose parameters used in logistic regression-based models (eg, mean doses for the parotid and submandibular glands). Of the 3 DL networks tested, the DCNN architecture performed slightly better than the other DL model types. The most important model input was the dose distribution (Fig. 3). Additionally, the observed relationship between the dose to the parotid glands and the DL risk predictions (Fig. 2) enhanced the reliability of the DL NTCP model. In line with this, the attention maps—illustrating the regions the model focuses on—showed a high focus on the parotid and submandibular glands (Fig. 4).

Achieving superior performance with DL models compared with logistic regression-based models can be challenging without a large patient cohort, because the effectiveness of DL models depends on having access to large data sets.^34^ Our results demonstrate that improvement was achieved with 759 patients for training and cross-validation. Further performance improvements could be achieved by increasing the data variation and size, unlocking the true potential of DL models.

Although the DL models performed well in the independent test set from the same institute, they did not perform well in external validation (AUC, 0.62–0.63). This result can be partially explained by variations in cohort composition or batch effects. For example, the external cohort consisted of 90% of patients diagnosed with oropharyngeal cancer, in contrast to 39% in the training and cross-validation sets (Table 1). Additionally, the 2 institutes differed in their endpoint assessment methods (4-point vs 11-point scale). However, it did not explain why the dose distribution did not add to the DL performance in the external cohort (Fig. 3). By retraining the model weights on a limited number of external patients (ie, transfer learning), the DL model again showed better performance than the reference model (which was also refitted for a fair comparison) in the external data set, and the DL dose dependency was restored. Transfer learning on 100 external patients showed potential for DL models (AUC_external_, 0.65–0.66 [0.56–0.74]) to surpass the performance of the reference model (AUC_external_, 0.64 [0.55–0.72]). A larger external cohort for transfer learning increasingly improved the DL performance. Because the difference between the institutes was overcome with minor model adaptation, transfer learning may be crucial if the DL model is presented with structurally different data. In other words, transfer learning presented a practical strategy to improve the generalizability of a DL-based NTCP model in a new clinical setting even if limited data are available.

The attention maps highlighted the important regions in the DL input for predicting Xer_12m_ per patient (Fig. 4). The attention maps illustrated that the salivary glands—both the parotid and submandibular glands—were important in predicting Xer_12m_, which is consistent with the variable of the salivary glands’ mean dose that is (nearly always) included in conventional NTCP models.^8,14,15^ More specifically, particularly for oropharyngeal cancer patients, high attention (red color shade in Fig. 4) was located posteriorly adjacent to the mandible in the parotid glands. This region resembled the region where the major salivary duct is bifurcated, which has been identified as the stem cell-rich region of the parotid glands by van Luijk et al.^33,35^ They demonstrated that the radiation dose delivered to structures containing parotid glands’ stem cells was a critical determinant of parotid glands’ dysfunction following radiation therapy. We consider it an interesting finding that the high-attention regions are consistent with the previously defined stem cell regions.

This study builds on and validates the work by Men et al,^17^ affirming that DL can improve the prediction of late xerostomia compared with the logistic regression model. Addressing Men et al^17^ suggestions, our research introduced clinical variables, conducted on a large cohort of 1208 patients incorporating recent cases up to 2021, reflecting contemporary radiation therapy protocols including proton therapy. Moreover, we used a conventional xerostomia NTCP model^8^ as a reference, offering a fairer and more challenging comparison with the DL-based NTCP model. Consistent evaluation metrics, including AUC, Brier score, Nagelkerke R^2^, and calibration plots, align with conventional NTCP model development standards,^36^ thereby showing a justified superior performance of the DL models in the independent test set. Methodologically for robustness purposes, this study introduced a reproducible data preprocessing procedure with an automated bounding box cropping algorithm (Supplementary Material E2), 10-fold stratified cross-validation, external validation combined with transfer learning, testing the impact of CT metal artifacts, and exploring attention maps—extending beyond individual feature maps. Collectively, these contributions enhance the understanding and applicability of late xerostomia DL-based NTCP models, setting a new benchmark for future investigations in the field to embark on clinical implementation.

Our results demonstrate that excluding CTs with metal artifacts may improve the DL model performance (Fig. 3b). Compared with all patients, excluding patients with CTs with a heavy metal artifact or with any metal artifact improved the model performance from AUC_test_, 0.79 [0.71–0.86] to AUC_test_, 0.81 [0.66–0.96] and from AUC_external_, 0.63 [0.55–0.70] to AUC_external_, 0.68 [0.51–0.85]. On one hand, this result suggests that a data set containing more (extreme) CT metal artifacts (eg, external set) could benefit more from removing metal artifacts. On the other hand, the impact of CT scans on the prediction performance was minimal when segmentations were supplied on both the internal and external set (Fig. 3a), showing that including either input modality with the dose distribution was sufficient for adequately predicting Xer_12m_.

The main limitation of this study is that although the developed models performed well in the internal set, they were not generalizable to the external set (without transfer learning). To mitigate this issue, it may be necessary to artificially make dose distributions, CT imaging, and OAR contouring of the external set consistent with those of the internal set, train the models on a large multicenter data set, or correct for batch effects, which could be challenging. Differences in scoring systems and data acquisition among medical centers may require different model parameter values, potentially impacting predictions when applying the model to an external cohort. This notion is supported by findings in Mavroidis et al^7^ and Roesink et al.^5^ Furthermore, the current mapping of the 11-point MD Anderson Symptom Inventory-Head and Neck Module score to a 4-point Likert scale may introduce inconsistencies in defining and classifying moderate-to-severe xerostomia between cohorts, potentially contributing to the observed performance drop in the external cohort. The performance decrease of the DL models compared with the reference model could be attributed to the utilization of more complex data (3D vs 1D). Regardless, the presented DL models should—in their current state—not be used without a thorough evaluation or transfer learning strategy.

Other limitations include the lack of exploration of alternative methods for incorporating clinical variables, which may limit the DL prediction performance, and the inability to directly compare model performances before and after excluding patients with CT metal artifacts because of differences in the composition of the evaluation set.

Suggestions for future work are collecting a large multicenter data set and exploring conversion methods to unify different scoring systems, investigating time-series data (eg, xerostomia at multiple time points), deforming the patient’s 3D data to a reference patient to decrease the variation that the DL model faces, exploring different methods for incorporating clinical variables, and reducing CT scans’ metal artifacts via style transfer to increase the uniformity of the input data.

## Conclusion

The DL-based NTCP models showed improvement in predicting xerostomia when evaluated on the never-seen-by-the-model test set compared with the reference NTCP model. The results suggest that xerostomia prediction can be improved by using the entire 3D dose distribution, CT scans, OAR segmentations, and clinical variables. Unfortunately, it showed that the reference NTCP model performed better than the DL models when tested on an external validation cohort from another center. Although transfer learning improved the DL model’s performance, it suggests the need for a multicenter data set for generalizing DL-based NTCP model development.

## Supplementary Material

Supplementary material

## Figures and Tables

**Table 1:** Patient demographics for the full study cohort and training and cross-validation, independent test, and external validation set separately

	Training		Independent test		External validation		P value
Total	759		138		311		
*Gender (%)							<.001
Female	186	(24)	38	(28)	29	(9)	
Male	573	(76)	100	(72)	282	(91)	
*Age (mean [SD])	64	(10)	64	(11)	60	(9)	<.001
Tumor site (%)							<.001
Hypopharynx	55	(7)	14	(10)	0	(0)	
Larynx	342	(45)	63	(46)	0	(0)	
Nasopharynx	37	(5)	7	(5)	0	(0)	
Oral cavity	30	(4)	9	(6)	0	(0)	
Oropharynx	295	(39)	45	(33)	281	(90)	
Unknown primary	0	(0)	0	(0)	30	(10)	
T stage (%)							<.001
T0	5	(1)	2	(1)	30	(10)	
T1	150	(20)	27	(20)	97	(31)	
T2	231	(30)	39	(28)	103	(33)	
T3	183	(24)	35	(25)	43	(14)	
T4	190	(25)	34	(25)	37	(12)	
Unknown	0	(0)	1	(1)	1	(0)	
N stage (%)							<.001
N0	368	(48)	64	(46)	21	(7)	
N1	72	(10)	18	(13)	109	(35)	
N2	298	(39)	54	(39)	174	(56)	
N3	20	(3)	2	(1)	5	(2)	
Unknown	1	(0)	0	(0)	2	(1)	
P16 HPV (%)							<.001
Negative	162	(21)	29	(21)	16	(5)	
Positive	143	(19)	23	(17)	239	(77)	
Unknown	454	(60)	86	(62)	54	(18)	
Technique (%)							<.001
3D-CRT	63	(8)	8	(6)	4	(1)	
IMPT	85	(11)	15	(11)	54	(17)	
IMRT	371	(49)	69	(50)	63	(20)	
VMAT	240	(32)	46	(33)	190	(61)	
CT with contrast (%)							<.001
No	144	(19)	24	(17)	311	(100)	
Yes	615	(81)	114	(83)	0	(0)	
CT metal artifact (%)							<.001
None	379	(50)	72	(52)	48	(15)	
Little/medium	165	(22)	29	(21)	130	(42)	
Heavy	215	(28)	37	(27)	133	(43)	
Xerostomia at 12 mo (%)							.002
0 (None-little)	490	(65)	89	(64)	231	(74)	
1 (Moderate-to-severe)	269	(35)	49	(36)	80	(26)	
*Baseline xerostomia (%)							.018
0 (None-little)	695	(92)	126	(91)	298	(96)	
1 (Moderate-to-severe)	64	(8)	12	(9)	13	(4)	

*Abbreviations:* 3D-CRT = 3-dimensional conformal radiation therapy; HPV = human papillomavirus; IMPT = intensity modulated proton therapy; IMRT = intensity modulated radiation therapy; SD = standard deviation; VMAT = volumetric-modulated arc therapy.

* The difference in patient cohorts were tested by Pearson’s *χ*^2^ test. Included as clinical variables in the deep learning models.

**Table 2:** Results of the 10-fold cross-validation (N = 759), independent test set (N = 138), external validation (N = 311), and external validation after transfer learning (N = 211) for each NTCP model

	Cohort	Reference	DCNN	EfficientNetV2-S	ResNet
AUC (95% CI)	Cross-validation	0.75 (0.72–0.78)	**0.79 (0.76–0.82)**	0.78 (0.75–0.81)	0.78 (0.75–0.81)
	Independent test*	0.74 (0.73–0.75)	**0.79 (0.77–0.81)**	0.78 (0.76–0.80)	0.78 (0.76–0.80)
	External validation*	**0.66 (0.63–0.69)**	0.63 (0.60–0.66)	0.63 (0.60–0.66)	0.62 (0.59–0.65)
	External validation after transfer learning*	0.64 (0.63–0.65)	**0.66 (0.63–0.69)**	0.65 (0.62–0.68)	**0.66 (0.63–0.69)**
Brier score	Cross-validation	0.20	**0.18**	**0.18**	**0.18**
	Independent test*	0.20	**0.18**	**0.18**	**0.18**
	External validation*	**0.20**	**0.20**	0.21	**0.20**
	External validation after transfer learning*	0.20	**0.19**	**0.19**	**0.19**
R^2^	Cross-validation	0.27	0.27	0.25	**0.28**
	Independent test*	0.20	**0.27**	0.26	0.26
	External validation*	−**0.05**	−0.07	−0.13	−0.10
	External validation after transfer learning*	0.06	0.07	0.06	**0.08**

*Abbreviations:* AUC = area under the receiver operating characteristic curve; R^2^ = Nagelkerke’s R^2^; DCNN = deep convolutional neural network; ResNet = residual neural network.

* Ensemble model.

## Data Availability

Research data are stored in an institutional repository and will be shared on request to the corresponding author.

## References

[1] Langendijk JA, Lambin P, De Ruysscher D, Widder J, Bos M, Verheij M. Selection of patients for radiotherapy with protons aiming at reduction of side effects: the model-based approach. Radiother Oncol 2013;107:267–273. 23759662 10.1016/j.radonc.2013.05.007

[2] Langendijk JA, Doornaert P, Verdonck-de Leeuw IM, Leemans CR, Aaronson NK, Slotman BJ. Impact of late treatment-related toxicity on quality of life among patients with head and neck cancer treated with radiotherapy. J Clin Oncol 2008;26:3770–3776. 18669465 10.1200/JCO.2007.14.6647

[3] Vissink A, van Luijk P, Langendijk JA, Coppes RP. Current ideas to reduce or salvage radiation damage to salivary glands. Oral Dis 2015;21:e1–e10. 24581290 10.1111/odi.12222

[4] Houweling AC, Philippens MEP, Dijkema T, A comparison of dose-response models for the parotid gland in a large group of head-and-neck cancer patients. Int J Radiat Oncol Biol Phys 2010;76:1259–1265. 20005639 10.1016/j.ijrobp.2009.07.1685

[5] Roesink JM, Moerland MA, Battermann JJ, Hordijk GJ, Terhaard CHJ. Quantitative dose-volume response analysis of changes in parotid gland function after radiotherapy in the head-and-neck region. Int J Radiat Oncol Biol Phys 2001;51:938–946. 11704314 10.1016/s0360-3016(01)01717-5

[6] Deasy JO, Moiseenko V, Marks L, Chao KSC, Nam J, Eisbruch A. Radiotherapy dose-volume effects on salivary gland function. Int J Radiat Oncol Biol Phys 2010;76:S58–S63. 20171519 10.1016/j.ijrobp.2009.06.090 PMC4041494

[7] Mavroidis P, Price A, Fried D, Dose-volume toxicity modeling for de-intensified chemo-radiation therapy for HPV-positive oropharynx cancer. Radiother Oncol 2017;124:240–247. 28712533 10.1016/j.radonc.2017.06.020

[8] Van den Bosch L, van der Schaaf A, van der Laan HP, Comprehensive toxicity risk profiling in radiation therapy for head and neck cancer: a new concept for individually optimised treatment. Radiother Oncol 2021;157:147–154. 33545258 10.1016/j.radonc.2021.01.024

[9] Beetz I, Schilstra C, Burlage FR, Development of NTCP models for head and neck cancer patients treated with three-dimensional conformal radiotherapy for xerostomia and sticky saliva the role of dosimetric and clinical factors. Radiother Oncol 2012;105:86–93. 21632133 10.1016/j.radonc.2011.05.010

[10] Samant P, de Ruysscher D, Hoebers F, Machine learning for normal tissue complication probability prediction: predictive power with versatility and easy implementation. Clin Transl Radiat Oncol 2023;39 100595. 36880063 10.1016/j.ctro.2023.100595 PMC9984444

[11] Guo Y, Jiang W, Lakshminarayanan P, Spatial radiation dose influence on xerostomia recovery and its comparison to acute incidence in patients with head and neck cancer. Adv Radiat Oncol 2020;5:221–230. 32280822 10.1016/j.adro.2019.08.009 PMC7136646

[12] Han P, Lakshminarayanan P, Jiang W, Dose/volume histogram patterns in Salivary Gland subvolumes influence xerostomia injury and recovery. Sci Rep 2019;9:3616. 30837617 10.1038/s41598-019-40228-y PMC6401158

[13] Monti S, Palma G, D’Avino V, Voxel-based analysis unveils regional dose differences associated with radiation-induced morbidity in head and neck cancer patients. Sci Rep 2017;7:7220. 28775281 10.1038/s41598-017-07586-x PMC5543173

[14] Beetz I, Schilstra C, Van Der Schaaf A, NTCP models for patient-rated xerostomia and sticky saliva after treatment with intensity modulated radiotherapy for head and neck cancer: the role of dosimetric and clinical factors. Radiother Oncol 2012;105:101–106. 22516776 10.1016/j.radonc.2012.03.004

[15] van Dijk LV, Brouwer CL, van der Schaaf A, CT image biomarkers to improve patient-specific prediction of radiation-induced xerostomia and sticky saliva. Radiother Oncol 2017;122:35. 10.1016/j.radonc.2016.07.007 27459902

[16] Gabryś HS, Buettner F, Sterzing F, Hauswald H, Bangert M. Design and selection of machine learning methods using radiomics and dosiomics for normal tissue complication probability modeling of xerostomia. Front Oncol 2018;8:1–20. 29556480 10.3389/fonc.2018.00035 PMC5844945

[17] Men K, Geng H, Zhong H, Fan Y, Lin A, Xiao Y. A deep learning model for predicting xerostomia due to radiation therapy for head and neck squamous cell carcinoma in the RTOG 0522 clinical trial. Int J Radiat Oncol Biol Phys 2019;105:440–447. 31201897 10.1016/j.ijrobp.2019.06.009 PMC6732004

[18] Christianen MEMC, Langendijk JA, Westerlaan HE, van de Water TA, Bijl HP. Delineation of organs at risk involved in swallowing for radiotherapy treatment planning. Radiother Oncol 2011;101:394–402. 21664711 10.1016/j.radonc.2011.05.015

[19] van der Laan HP, Gawryszuk A, Christianen MEMC, Swallowing-sparing intensity-modulated radiotherapy for head and neck cancer patients: treatment planning optimization and clinical introduction. Radiother Oncol 2013;107:282–287. 23742959 10.1016/j.radonc.2013.05.004

[20] Vergeer MR, Doornaert PAH, Rietveld DHF, Leemans CR, Slotman BJ, Langendijk JA. Intensity-modulated radiotherapy reduces radiation-induced morbidity and improves health-related quality of life: results of a nonrandomized prospective study using a standardized follow-up program. Int J Radiat Oncol Biol Phys 2009;74:1–8. 19111400 10.1016/j.ijrobp.2008.07.059

[21] Rosenthal DI, Mendoza TR, Chambers MS, Measuring head and neck cancer symptom burden: the development and validation of the M. D. Anderson symptom inventory, head and neck module. Head Neck 2007;29:923–931. 17358040 10.1002/hed.20602

[22] Krizhevsky A, Sutskever I, Hinton GE. ImageNet classification with deep convolutional neural networks. Adv Neural Inf Process Syst 2012;25.

[23] Tan M, Le Qv. EfficientNetV2: Smaller Models and Faster Training; 2021.

[24] He K, Zhang X, Ren S, Sun J. Deep residual learning for image recognition. In: Proceedings of the IEEE Computer Society Conference on Computer Vision and Pattern Recognition 2016. 770–778.

[25] Cardoso MJ, Li W, Brown R, MONAI: an open-source framework for deep learning in healthcare; 2022. 10.48550/arXiv.2211.02701.

[26] Paszke A, Gross S, Massa F, PyTorch: an imperative style, high-performance deep learning library; 2019. 10.48550/arXiv.1912.01703.

[27] Nagelkerke NJD. A note on a general definition of the coefficient of determination. Biometrika 1991;78:691–692.

[28] Zhou B, Khosla A, Lapedriza A, Oliva A, Torralba A. Learning deep features for discriminative localization. 2016 IEEE Conference on Computer Vision and Pattern Recognition. Las Vegas, NV; 2015:2921–2929. 27–30 June 2016.

[29] Chattopadhay A, Sarkar A, Howlader P, Balasubramanian VN. Grad-CAM++: generalized gradient-based visual explanations for deep convolutional networks. In: Proceedings of the 2018 IEEE Winter Conference on Applications of Computer Vision, WACV 2018. 839–847.

[30] Gotkowski K, Gonzalez C, Bucher A, Mukhopadhyay A. M3d-CAM: a PyTorch library to generate 3D data attention maps for medical deep learning. DeepAI; 2020. Accessed January 21, 2022. https://deepai.org/publication/m3d-cam-a-pytorch-library-to-generate-3d-data-attention-maps-for-medical-deep-learning.

[31] Luo L, Xiong Y, Liu Y, Sun X. Adaptive gradient methods with dynamic bound of learning rate. 7th International Conference on Learning Representations. 2019, ICLR; 2019:1–19.

[32] Loshchilov I, Hutter F. SGDR: stochastic gradient descent with warm restarts; 2016. 10.48550/arXiv.1608.03983.

[33] van Luijk P, Pringle S, Deasy JO, Sparing the region of the salivary gland containing stem cells preserves saliva production after radiotherapy for head and neck cancer. Sci Transl Med 2015;7:305ra147. 10.1126/scitranslmed.aac4441 PMC4964284 26378247

[34] Alzubaidi L, Zhang J, Humaidi AJ, Review of deep learning: concepts, CNN architectures, challenges, applications, future directions. J Big Data 2021;8:53. 33816053 10.1186/s40537-021-00444-8 PMC8010506

[35] Fried DV, Zhu T, Das SK, Prospective assessment of sparing the parotid ducts via MRI sialography for reducing patient reported xerostomia. Radiother Oncol 2022;172:42–49. 35537605 10.1016/j.radonc.2022.05.001

[36] Van den Bosch L, Schuit E, van der Laan HP, Key challenges in normal tissue complication probability model development and validation: towards a comprehensive strategy. Radiother Oncol 2020;148: 151–156. 32388149 10.1016/j.radonc.2020.04.012

