# Supplementary material for: Three-Dimensional Deep Learning Normal Tissue Complication Probability Model to Predict Late Xerostomia in Patients With Head and Neck Cancer
Source: Int J Radiat Oncol Biol Phys. Author manuscript; Available in PMC 2025 Jan 1. (PMC11646177; doi:10.1016/j.ijrobp.2024.07.2334)
Supplement: Supplementary material [file NIHMS2034725-supplement-Supplementary_material.pdf]

Supplementary material

S1 – Reference model

The reference model was a multivariable logistic regression-based NTCP model that used the sum square root of the mean dose to parotid glands, the mean dose to submandibular glands, and baseline toxicity values, as in [1]. The normal tissue complication probability (NTCP) was calculated by the model as follows:

$$NTCP = \frac{1}{1+e^{-y}},$$

where y was obtained from combining two separately fitted submodels:

$$y^{(1)} = intercept^{(1)} + \beta_1^{(1)} * a + \beta_2^{(1)} * c + \beta_3^{(1)} * d,$$
$$y^{(2)} = intercept^{(2)} + \beta_1^{(2)} * b + \beta_2^{(2)} * c + \beta_3^{(2)} * d,$$
$$y = \frac{1}{2}(y^{(1)} + y^{(2)}),$$

where *a* = mean dose to submandibular glands, *b* = sum square root of the mean dose to parotid glands, *c* = baseline toxicity 'a little', and *d* = baseline toxicity 'moderate-to-severe'.

Table 1 lists the coefficients of the trained reference logistic regression-based NTCP model from [1], the average of internal cross-validation refits, and the average of external cross-validation refits.

Table 1. Trained coefficients of the reference logistic regression-based NTCP model.

Model predictors	Original	Internal refit	External refit
Intercept	-2.9032	-2.8078	-4.1600
Mean dose submandibular glands	0.0193	0.0162	0.0190
Adjusted mean dose parotid glands*	0.1054	0.0860	0.2066
Baseline toxicity 'a little'	0.5234	1.4375	0.0867
Baseline toxicity 'moderate-to-severe'	1.2763	1.8535	1.0102

* sum square root = $\sqrt{mean\ dose\ left\ parotid\ gland}$ + $\sqrt{mean\ dose\ right\ parotid\ gland}$.

S2 – Data pre-processing, augmentation, and input

The planning CT scans were obtained from different planning systems (MDS Nordion, SIEMENS, and Siemens Healthineers). An overview of the CT scanner parameters is presented in Table 2. Organs-at-risk were delineated according to the CT-based delineation guidelines [2]. Segmentations of the following organs-at-risk were included: buccal mucosa, oral cavity, parotid glands, and submandibular glands. All imaging modalities were stored in standardized DICOM-RT format.

Table 2. InstituteX_EU CT scanning parameters

Manufacturer	SIEMENS
**Number of patients**	897
**Model name(s)**	Biograph 64, Sensation 16, Sensation Open, SOMATOM Definition AS
**Pixel spacing (mm)**	0.7 – 1.6
**Slice thickness (mm)**	2.0 – 4.0
**KVP**	80 – 140

Due to anonymization, the InstituteX_US CT scanning parameters were unavailable to us.

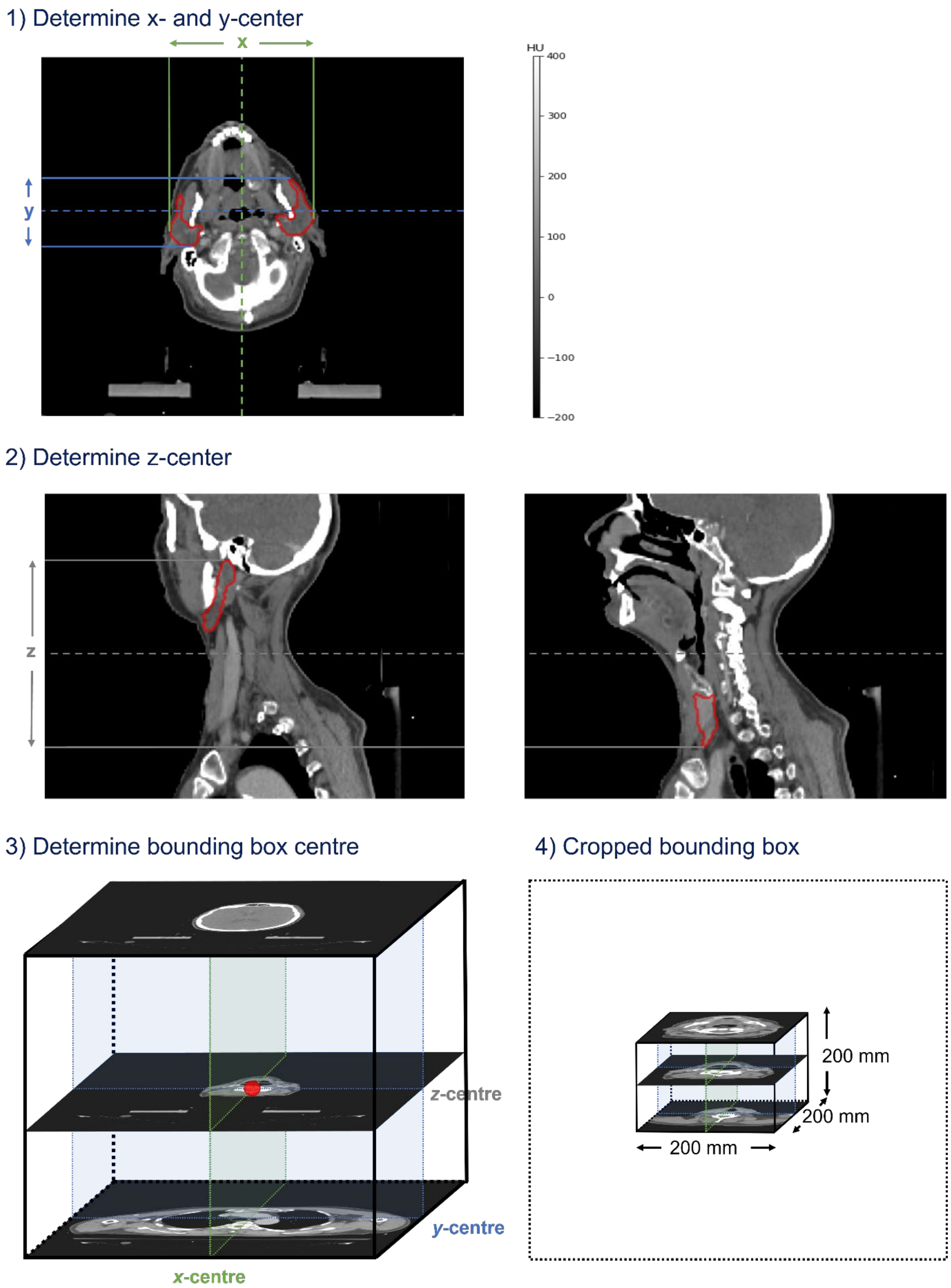

Figure 1. 1) For the *x*-dimension, the upper and lower voxel index in the parotid and submandibular glands segmentation map was extracted, and the middle was set as the *x*-center voxel index. The voxels did not have to be on the slice. For the *y*-dimension, similarly, using only parotid glands. 2) For the *z*-dimension, the parotid glands, cricoid, and thyroid were used. 3) The (*x*, *y*, *z*)-center coordinate was used to determine the bounding box region. 4) A bounding box region of 200 × 200 × 200 mm^3 around the (*x*, *y*, *z*)-center coordinate was cropped to obtain the bounding box region.

For each patient, the 3D planned dose distribution, corresponding planning CT scans, and organ-at-risk segmentations were resampled to an isotropic voxel spacing of 2 × 2 × 2 mm^3, where each modality was merged in a different 3D array channel. As illustrated in Figure 1, to reduce background noise, a bounding box of 200 × 200 × 200 mm^3 (depth × height × width) around organs-at-risk was cropped, resulting in a 3-channel array with dimensions of 100 × 100 × 100. The dose and CT intensity values were expressed in centigray (cGy) and Hounsfield Unit (HU), respectively. As input for the DL models, the dose and CT intensity values were clipped between [0, 8000] cGy and [-200, 400] HU, respectively, and then normalized to [0, 1] as per

$$x_{dose,norm} = \frac{x_{dose}(cGy)}{8000},$$
$$x_{CT,norm} = \frac{x_{CT}(HU) - (-200)}{400 - (-200)} = \frac{x_{CT}(HU) + 200}{600},$$

The organ-at-risk segmentation has values in [0, 1] for organs-at-risk and value 0 for background. The baseline xerostomia score was categorized into ‘none’, ‘little’, and ‘moderate-to-severe’. Sex was dichotomized into ‘1’ for males and ‘0’ for females. Age was normalized to [0, 1] by dividing by 100.

Data augmentation was applied to effectively increase the size of the training dataset to improve the model’s generalizability [3,4]. The following random 3D data augmentation techniques were implemented, where s is the data augmentation strength hyperparameter:

- Cropping spatial size of 96 × 96 × 96.
- Horizontal flipping (x-dimension).
- Translating between [-7 * s, 7 * s] voxels.
- Zooming by a factor between [-0.07 * s, 0.07 * s].
- Rotating by a radius between [-7.5° * s, 7.5° * s] (for every slice the same).

Each data augmentation technique was applied independently with a probability of 0.5. The transformed data were further processed by the AugMix algorithm [3] using the same random data augmentation techniques.

S3 – DL architectures

The DL models consisted of a stack of consecutive blocks, followed by a fully-connected layer to which the baseline xerostomia score, sex, and age are concatenated. The DCNN used convolutional blocks, the EfficientNetV2-S [5] used Squeeze and Excitation, inverted residual, and Fused-MBConv blocks, all containing the Swish activation function [6], and the ResNet [7] used residual blocks (Figure 2).

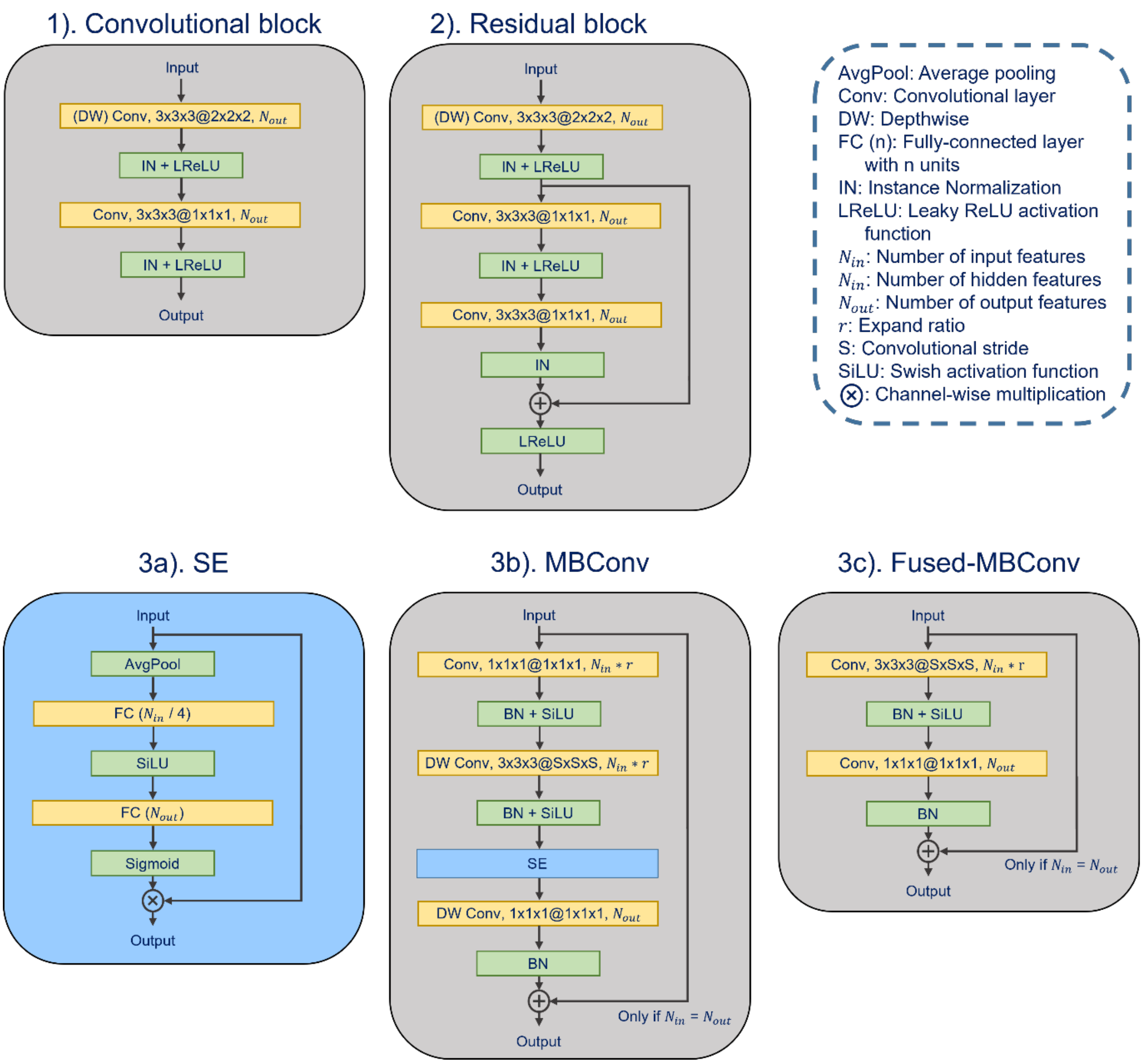

Figure 2. 1) Convolutional block used by DCNN; 2) residual block used by ResNet; 3a) Squeeze and Excitation (SE), 3b) inverted residual (MBConv), and 3c) Fused-MBConv blocks used by EfficientNetV2-S.

The blocks took 4D arrays as input and outputted a 4D array with the same resolution as the input or half of the input depth, height, and width. The output of a block was the input of the next block or layer. The first operator of the first Convolutional/Residual block used a depthwise convolution instead of a traditional convolution. In each model, the last block in the stack was followed by an average pooling or flattening layer, which was connected to a fully-connected layer before reaching the prediction layer. Baseline xerostomia score, sex, and age were concatenated to this fully-connected layer. The DCNN, EfficientNetV2-S, and ResNet had 140.1 thousand, 22.4 million, and 149 thousand trainable weight parameters, respectively.

Table 3. EfficientNetV2-S architecture, where MBConv and Fused-MBConv blocks are shown in Figure 2. Number 'k' in 'Conv, k × k × k' denotes the kernel size. In each stage, the convolution stride (S) only holds for the first layer of the corresponding block: subsequent layers use S = 1.

Stage	Operator	Convolution stride (S)	Expand ratio	Number of channels/units	Number of blocks/layers
1	Conv, 3 × 3 × 3	2	-	24	1
2	Fused-MBConv	1	1	24	2
3	Fused-MBConv	2	4	48	4
4	Fused-MBConv	2	4	64	4
5	MBConv	2	4	128	6
6	MBConv	1	6	160	9
7	MBConv	2	6	256	15
8	Conv, 1 × 1 × 1	1	-	1280	1
9	Average pooling	-	-	1280	1
10	Concatenate clinical variables	-	-	1285	1
11	Output	-	-	1	1

The DL model was trained for maximal 100 epochs with batch size eight and cross-entropy loss function in each cross-validation fold. The starting learning rate was 0.0001 and updated according to a scheduling scheme. Model weights at the epoch with the best validation score were saved during training. The training was stopped if the validation score did not improve for 20 consecutive epochs.

Extensive hyperparameter tuning was conducted to determine the optimal hyperparameter values. Hyperparameters were tuned in order of most to least expected impact on the model performance.

1. Model size:
    a. DCNN, ResNet:
        i. Number of blocks: 3, 4, 5, 6
        ii. Number of filters: 8, 16, 32, …, 256
        iii. Filter size: 2, 3, …, 9
        iv. Strides: 1, 2
        v. Number of fully-connected layers: 1, 2, 3, 4
        vi. Number of fully-connected units: 8, 16, 32, …, 256
    b. EfficientNetV2: S, M, L, XL
2. Initial learning rate: 1e-2, 1e-3, …, 1e-6
3. Optimizer: AccSGD [8], AdaBelief [9], AdaBound [10], Adam [11], AdaMod [12], Apollo [13], diffGrad [14], MADGRAD [15], NovoGrad [16], QHAdam [17], QHM [18], Radam [19], Ranger21 [20], RangerQH [17,18], RMSProp, SGD [21], SWATS [22], Yogi [23]
4. Learning rate scheduler:
    a. None
    b. Cosine with warm restarts T_0 [24]: 8, 16, 20, 32, 40, 64
    c. Exponential γ
5. Data augmentation strength: 1, 1.25, …, 4
6. Augmix strength: 1, 1.25, …, 4
7. Batch size: 1, 2, 4, 8, 16, 32
8. Dropout: 0, 0.05, …, 0.50
9. Label smoothing: 0, 0.1, 0.2
10. 3D to 1D flattening method: convolution, average pooling, max pooling
11. Use bias: True, False
12. Segmentation values: 0, 0.1, 0.25, 0.5, 0.75, 0.9, 1

Table 4 contains a comprehensive list of the training methodology and final selected hyperparameters for each DL model.

Table 4. Training methodology and selected hyperparameter values for each DL model. The training setting could be different for models that were trained after excluding patients with CT containing metal artifacts: * excluding metal artifact level 2, and ** excluding metal artifact levels 1 and 2.

Methodology	DCNN	EfficientNetV2-S	ResNet
**Maximum epochs**	100	100	100
**Batch size**	8	8	8
**Loss function**	Cross-entropy	Cross-entropy	Cross-entropy
Label smoothing	0	0	0
**Optimizer**	AdaBound	AdaBound	AdaBound | MADGRAD* | AdaBound**
Learning rate	1e-4	1e-4	1e-4
β_1	0.9	0.9	0.9
β_2	0.999	0.999	0.999
Weight decay	0.05	0	0.01
**Learning rate scheduler**	Cosine (T_0 = 40)	Cosine (T_0 = 40)	Cosine (T_0 = 40)
**Activation function**	Leaky ReLU (slope = 0.1)	Swish	Leaky ReLU (slope = 0.1)
**Weight initialisation**	Kaiming uniform [25]	Normal distribution (mean = 0, standard deviation = 2 / fan_out)	Kaiming uniform [25]
**Dropout probability**	0	0	0.25
**Data augmentation strength (s)**	1	3 | 2.5* | 2.25**	1 | 1.75* | 1**
**AugMix**			
Strength (s)	3	3 | 2.5* | 3**	2 | 1.75* | 3**
Width	3	3	3
Depth	1 – 3 (randomly)	1 – 3 (randomly)	1 – 3 (randomly)
**Segmentation values**			
Buccal mucosa	0.1	0.1	0.1
Oral cavity	0.1	0.1	0.1
Parotid glands	1	1	1
Submandibular glands	1	1	1

S4 – DL input modality dependency

The CT metal artifact level was assessed on a 3-point scale: 0 ('no metal artifact'), 1 ('little' and 'medium'), and 2 ('heavy'). A score of 0 was assigned if no metal artifact was visible in any slices, a score of 2 was assigned if a CT scan contained metal artifacts that affected a substantial area of the CT scan, and a score of 1 was assigned otherwise. This assessment was conducted by manually inspecting all slices containing voxels of the oral cavity, with multiple individuals participating to ensure consistency. Examples of each score are shown in Figure 3. Multiple people re-evaluated the scores for unification.

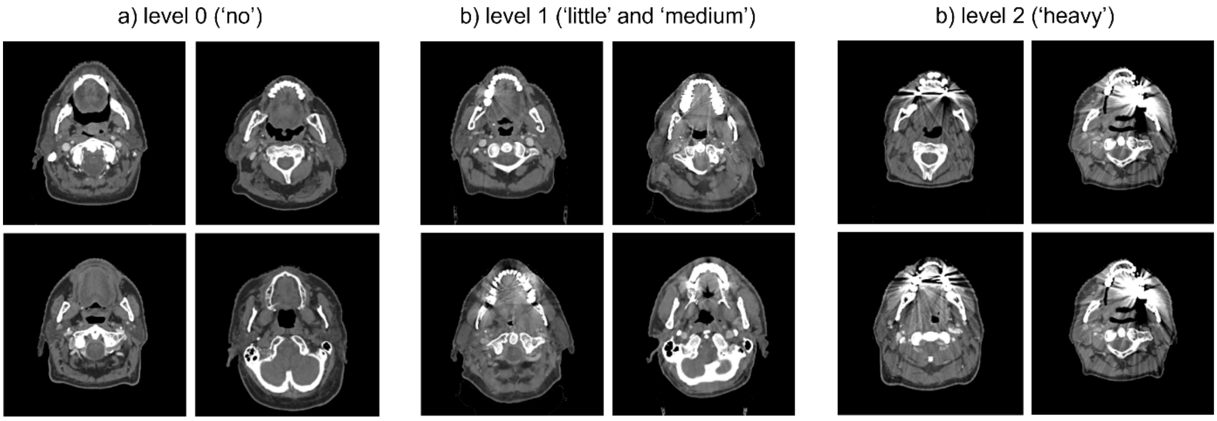

Figure 3. Slices of distinct patients with CT metal artifact a) level 0 ('no'); b) level 1 ('little' and 'medium'); c) level 2 ('heavy').

S5 – Looking inside the black box

Attention maps visualize which regions of the input image a DL model focuses on when making its prediction [26]. Moreover, attention maps highlight a DL model's visual features in input images. High (resp. low) attention values indicate that the input region has a large (resp. little) impact on the prediction value.

S6 – Calibration plots

Figure 4a. Independent test calibration plots of all models using ten bins. The red dashed line represents the fit of the bin's data points. The corresponding formula of the fit is shown in the left-top corner.

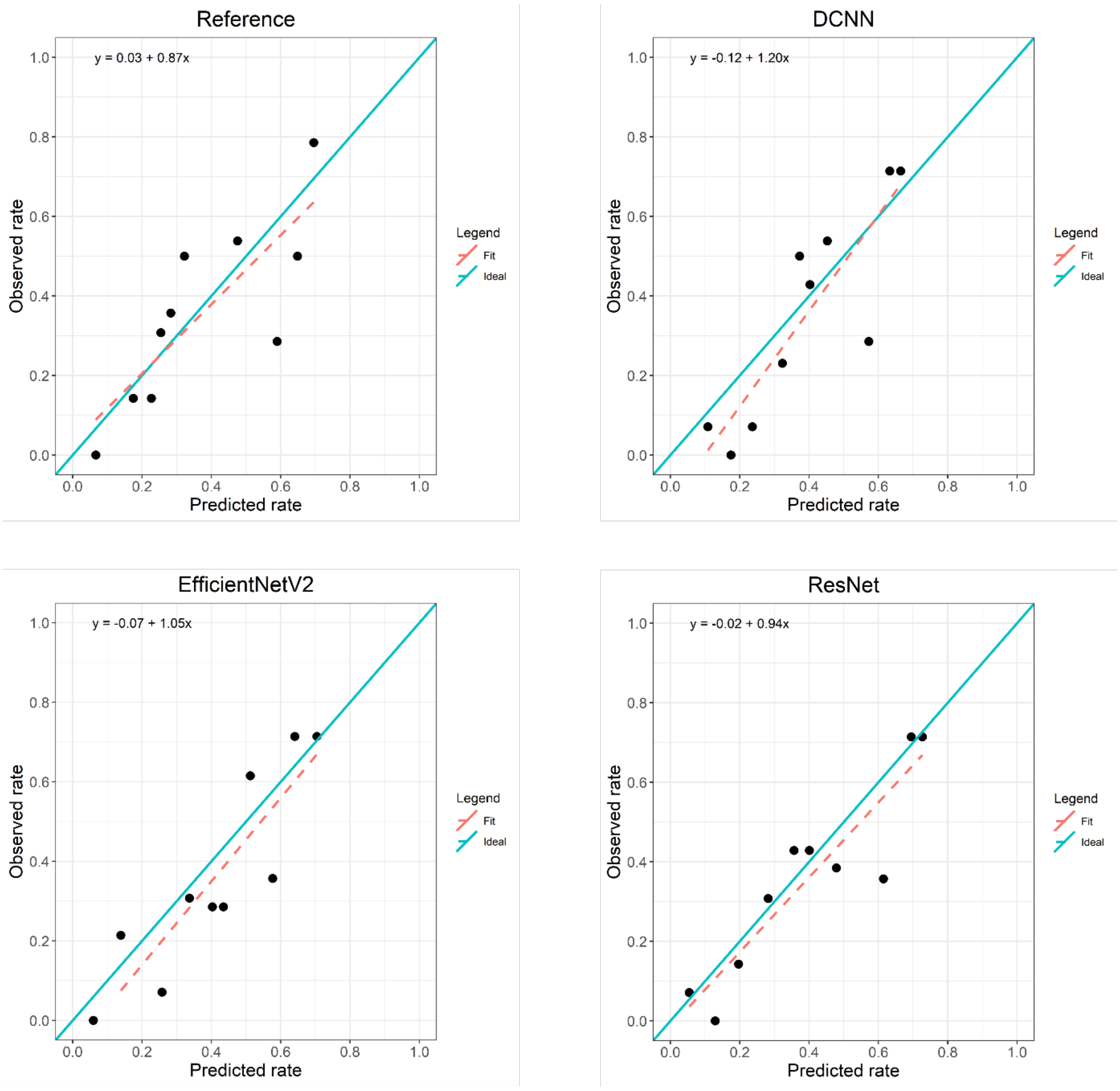

Figure 4b. External validation calibration plots of all models using ten bins. The red dashed line represents the fit of the bin's data points. The corresponding formula of the fit is shown in the left-top corner.

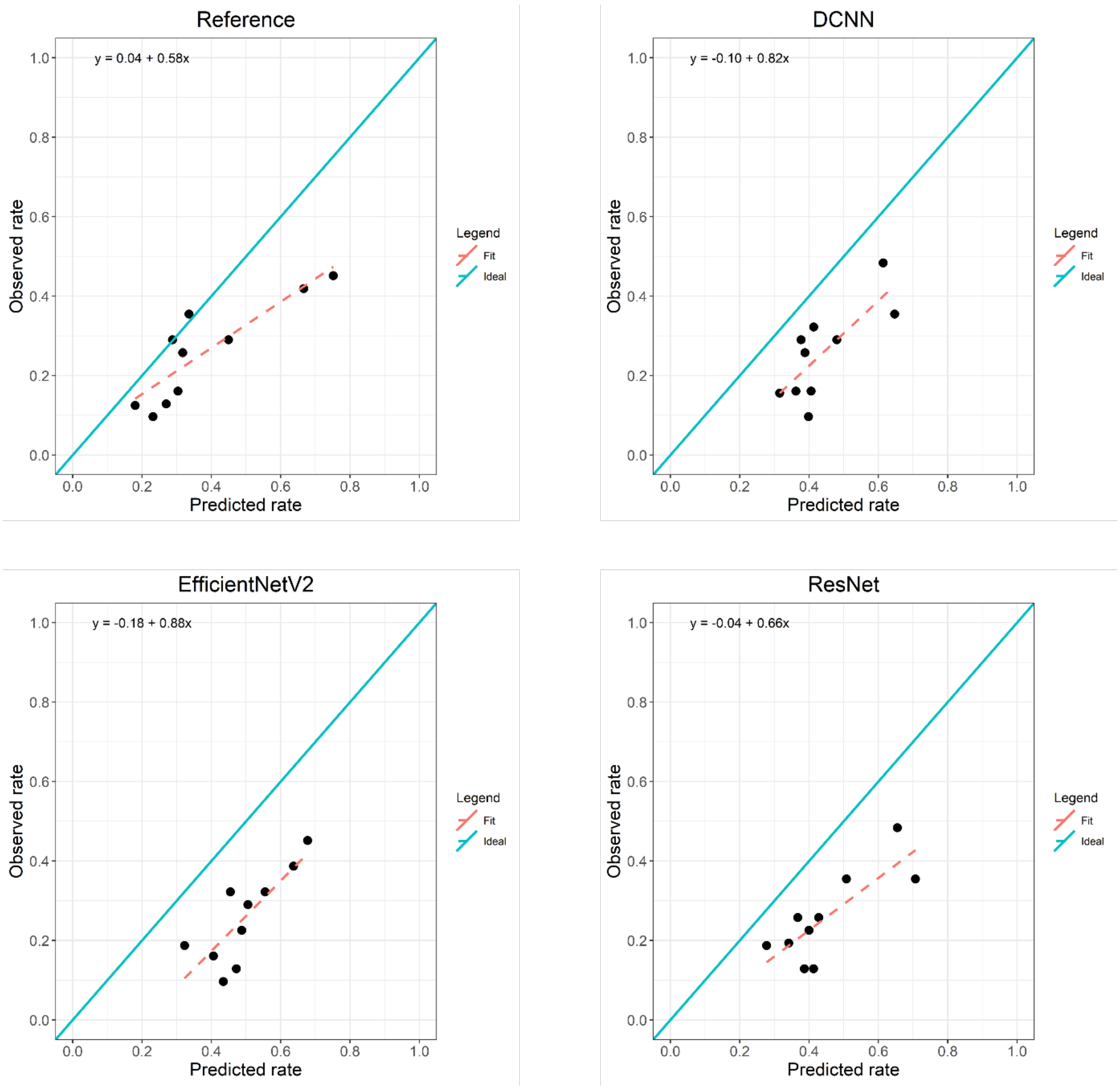

Figure 4c. External validation after transfer learning calibration plots of all models using ten bins. The red dashed line represents the fit of the bin's data points. The corresponding formula of the fit is shown in the left-top corner.

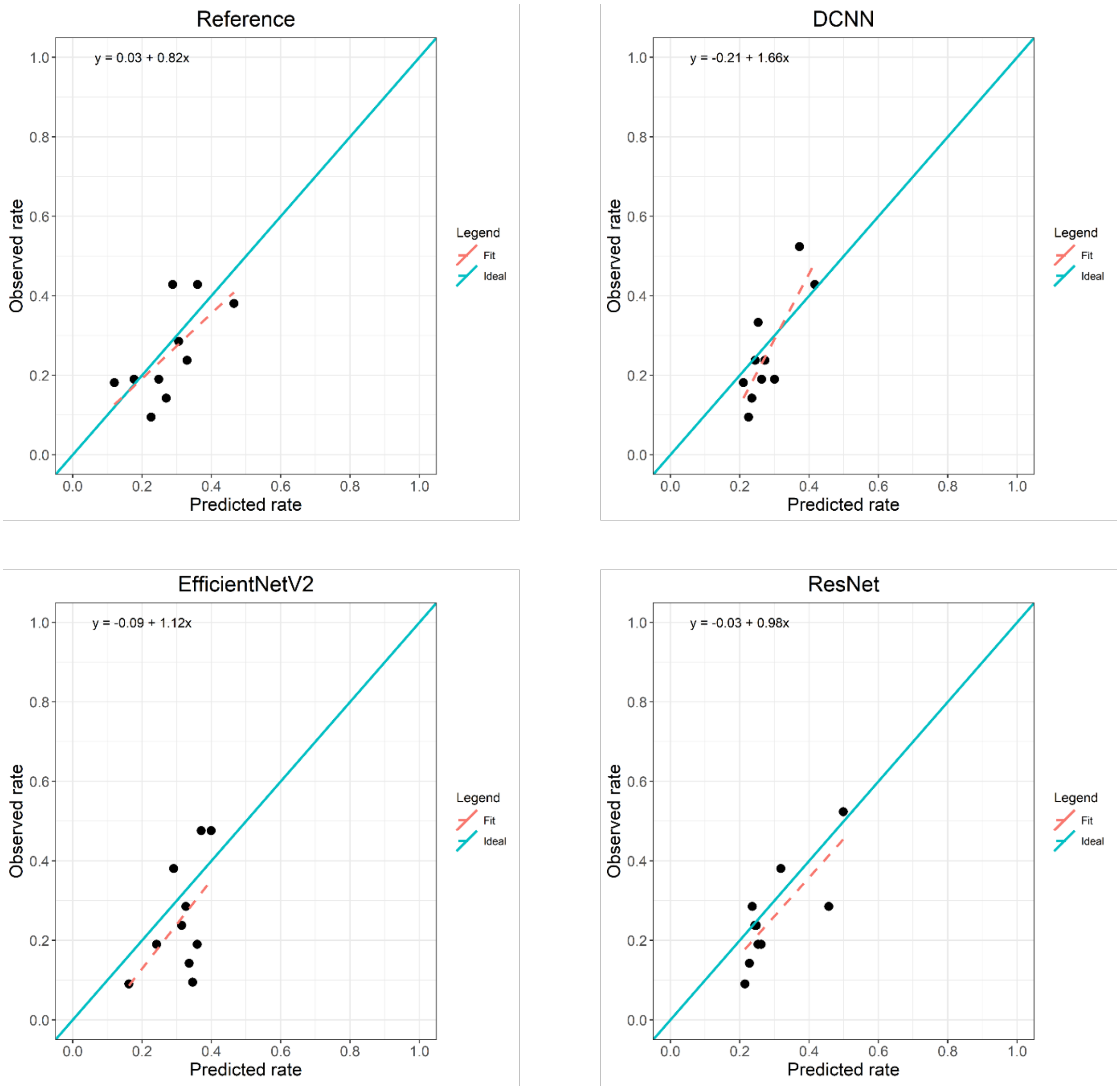

S7 – Transfer learning

Figure 5. DCNN’s test performance after transfer learning on different numbers of external (InstituteX_US) training samples (DeLong’s test *p*-value: 0.47 – 0.97).

Option 1

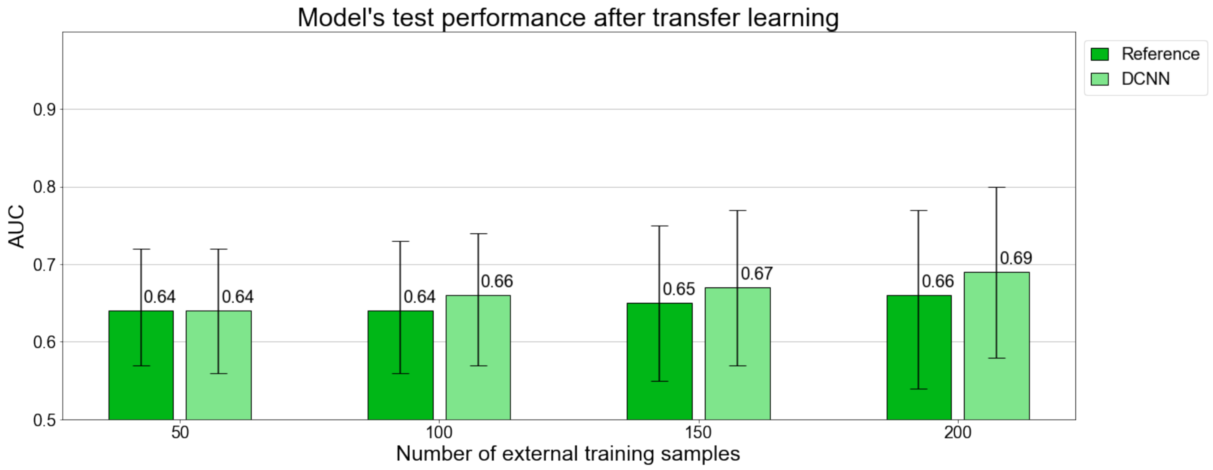

Option 2

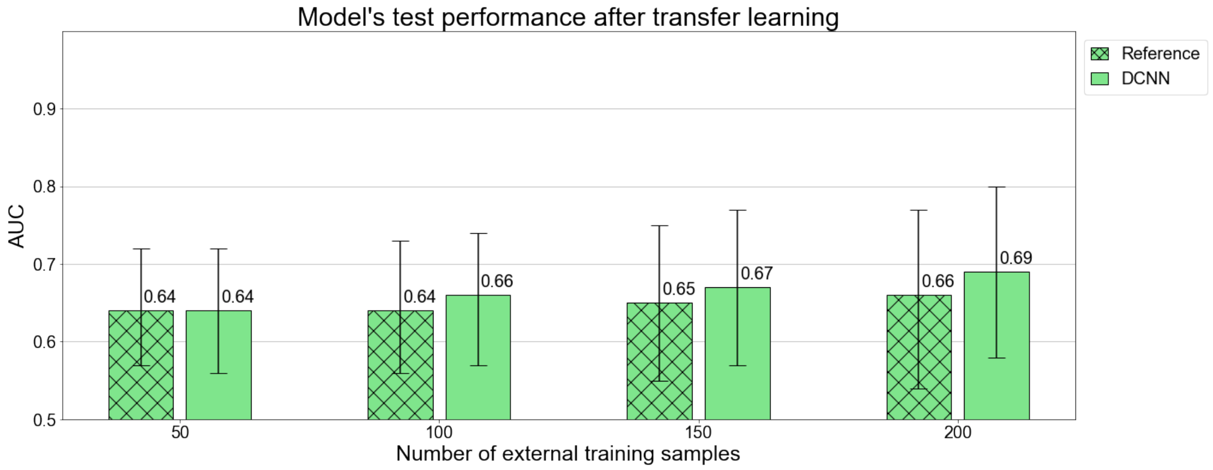

S8 – DL input modality dependency

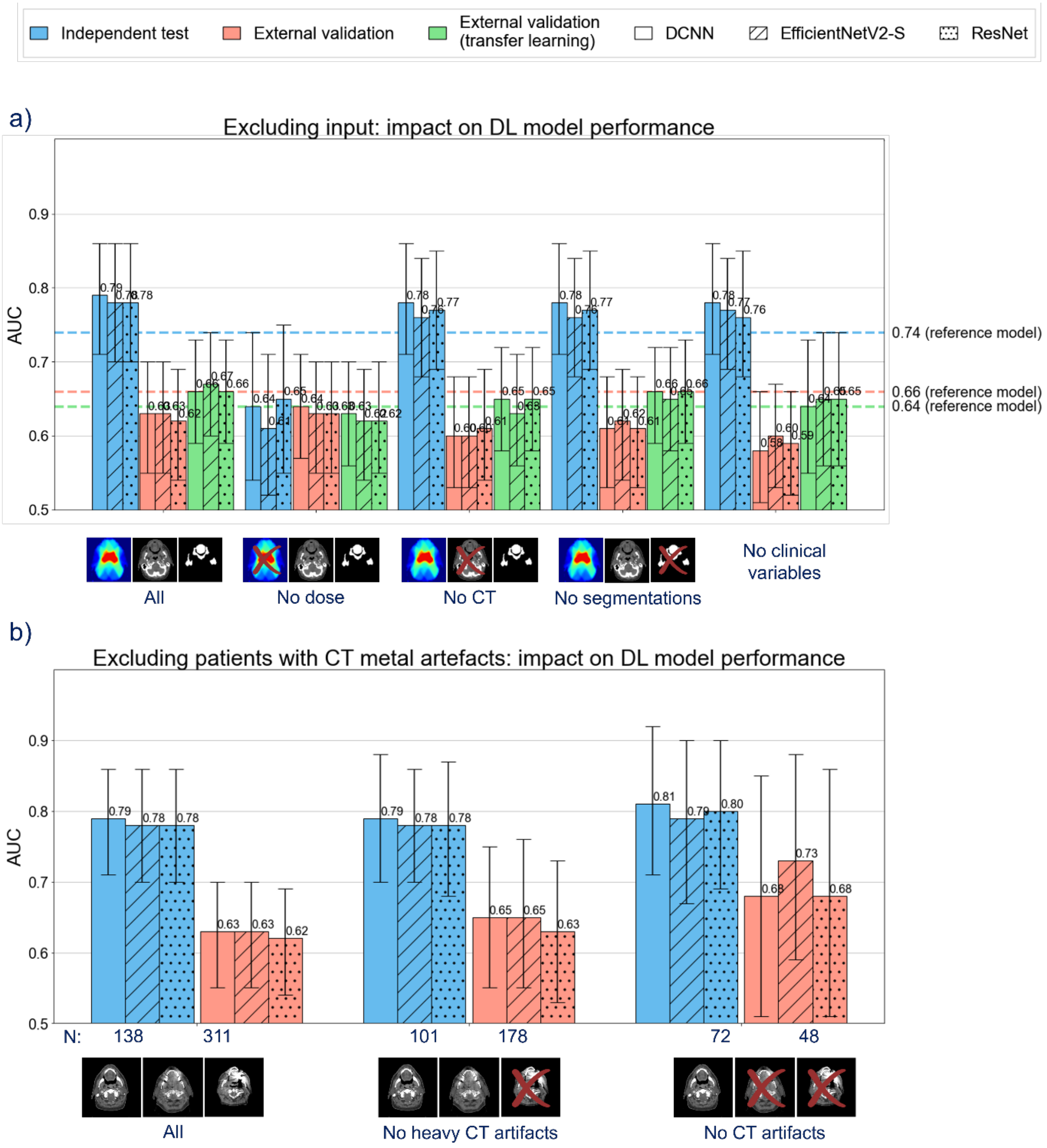

Figure 6. Model results of excluding a) input modality; and b) patients with metal artifact CT scans (where N denotes the number of patients of that data subset). The AUC value is shown inside the bar.

References

[1] Van den Bosch L, van der Schaaf A, van der Laan HP, Hoebers FJP, Wijers OB, van den Hoek JGM, et al. Comprehensive toxicity risk profiling in radiation therapy for head and neck cancer: A new concept for individually optimised treatment. Radiotherapy and Oncology 2021;157:147–54.

[2] Brouwer CL, Steenbakkers RJHM, Bourhis J, Budach W, Grau C, Grégoire V, et al. CT-based delineation of organs at risk in the head and neck region: DAHANCA, EORTC, GORTEC, HKNPCSG, NCIC CTG, NCRI, NRG Oncology and TROG consensus guidelines. Radiotherapy and Oncology 2015;117:83–90.

[3] Hendrycks D, Mu N, Cubuk ED, Zoph B, Gilmer J, Lakshminarayanan B. AugMix: A Simple Data Processing Method to Improve Robustness and Uncertainty 2019:1–15.

[4] Shorten C, Khoshgoftaar TM. A survey on Image Data Augmentation for Deep Learning. J Big Data 2019;6.

[5] Tan M, Le Q v. EfficientNetV2: Smaller Models and Faster Training 2021.

[6] Ramachandran P, Zoph B, Le Q V. Searching for Activation Functions 2017.

[7] He K, Zhang X, Ren S, Sun J. Deep residual learning for image recognition. Proceedings of the IEEE Computer Society Conference on Computer Vision and Pattern Recognition 2016;2016-Decem:770–8.

[8] Jain P, Kakade SM, Kidambi R, Netrapalli P, Sidford A. Accelerating Stochastic Gradient Descent For Least Squares Regression 2017.

[9] Zhuang J, Tang T, Ding Y, Tatikonda S, Dvornek N, Papademetris X, et al. AdaBelief Optimizer: Adapting Stepsizes by the Belief in Observed Gradients 2020.

[10] Luo L, Xiong Y, Liu Y, Sun X. Adaptive gradient methods with dynamic bound of learning rate. 7th International Conference on Learning Representations, ICLR 2019 2019:1–19.

[11] Kingma DP, Ba J. Adam: A Method for Stochastic Optimization 2014.

[12] Ding J, Ren X, Luo R, Sun X. An Adaptive and Momental Bound Method for Stochastic Learning 2019.

[13] Ma X. Apollo: An Adaptive Parameter-wise Diagonal Quasi-Newton Method for Nonconvex Stochastic Optimization 2020.

[14] Dubey SR, Chakraborty S, Roy SK, Mukherjee S, Singh SK, Chaudhuri BB. diffGrad: An Optimization Method for Convolutional Neural Networks 2019.

[15] Defazio A, Jelassi S. Adaptivity without Compromise: A Momentumized, Adaptive, Dual Averaged Gradient Method for Stochastic Optimization 2021.

[16] Ginsburg B, Castonguay P, Hrinchuk O, Kuchaiev O, Lavrukhin V, Leary R, et al. Stochastic Gradient Methods with Layer-wise Adaptive Moments for Training of Deep Networks 2019.

[17] Ma J, Yarats D. Quasi-hyperbolic momentum and Adam for deep learning 2018.

[18] Zhang MR, Lucas J, Hinton G, Ba J. Lookahead Optimizer: k steps forward, 1 step back 2019.

[19] Liu L, Jiang H, He P, Chen W, Liu X, Gao J, et al. On the Variance of the Adaptive Learning Rate and Beyond 2019.

[20] Wright L, Demeure N. Ranger21: a synergistic deep learning optimizer 2021.

[21] Robbins H, Monro S. A Stochastic Approximation Method. Source: The Annals of Mathematical Statistics 1951;22:400–7.

[22] Keskar NS, Socher R. Improving Generalization Performance by Switching from Adam to SGD 2017.

[23] Zaheer M, Reddi SJ, Sachan D, Kale S, Research G, Kumar S. Adaptive Methods for Nonconvex Optimization. NIPS'18: Proceedings of the 32nd International Conference on Neural Information Processing Systems 2018:9815–25.

[24] Loshchilov I, Hutter F. SGDR: Stochastic Gradient Descent with Warm Restarts 2016.

[25] He K, Zhang X, Ren S, Sun J. Delving Deep into Rectifiers: Surpassing Human-Level Performance on ImageNet Classification 2015.

[26] Zhou B, Khosla A, Lapedriza A, Oliva A, Torralba A. Learning Deep Features for Discriminative Localization 2015.
